# The Impact of Body Mass Index (BMI) on Clinical Outcomes for Patients Receiving Systemic Anti-Cancer Therapies for Advanced Clear Cell Renal Carcinoma

**DOI:** 10.1177/10732748251317681

**Published:** 2025-03-17

**Authors:** John Greene, Zhe Wang, Benjamin H. L. Harris, David Dodwell, Simon R. Lord

**Affiliations:** 1 Nuffield Department of Population Health, 227951 University of Oxford, Oxford, UK; 2 Computational Biology and Integrative Genomics Lab, Department of Oncology, 360534 University of Oxford, Oxford, UK

**Keywords:** obesity, BMI, cancer

## Abstract

**Introduction:**

Obesity is a risk factor for the development of renal cell carcinoma (RCC), however observational studies have suggested patients with RCC receiving systemic anti-cancer therapy (SACT) and BMI ≥25 kg/m^2^ may have a better prognosis than patients with a normal or low BMI, a phenomenon often referred to as the obesity paradox.

**Methods:**

The impact of BMI on survival outcomes in patients with advanced clear cell RCC receiving SACT within the National Health Service (NHS) in England between 2010 and 2018 was investigated. A retrospective analysis was performed using the SACT dataset from NHS-England.

**Results:**

A total of 1034 patients were included. The majority of patients commenced treatment with oral SACT, pazopanib (53.3%) and sunitinib (43.7%). Median overall survival for patients with BMI ≤25 kg/m^2^ was 12.6 months (95% CI; 10.1-14.4) and 17.9 months (15.4-20.0) for patients with BMI ≥25 kg/m^2^ (*P* < .001). The association between BMI and improved survival was greatest in the first year of commencing SACT with the adjusted mortality rate of 68.9% for patients with BMI less than 25 kg/m^2^ compared to 48.6% for patients with BMI greater than 25 kg/m^2^ (rate ratio .77, .63 to .93).

**Conclusion:**

A high BMI compared to a normal or low BMI was associated with improved survival in patients with metastatic RCC who were predominantly treated with oral SACT. Improved survival in obese patients with advanced RCC may be associated with improved response to systemic targeted therapies.

## Plain Language Summary

This project reviewed the impact of an individual’s weight on the treatment they received for advanced kidney cancer.

## Introduction

Obesity is a chronic disease with increasing prevalence globally, leading to worsening morbidity and mortality and increasing demands on healthcare systems.^1-3^ Obesity can be defined by body mass index (BMI) measurement, which can be used as a surrogate measurement of excess visceral fat accumulation, with a normal BMI defined as between 18.5-24.9 kg/m^2^, a BMI greater than 25 kg/m^2^ as overweight, and a BMI greater than 30 kg/m^2^ characterised as obese.^4^ A number of different mechanisms have been described by which obesity may drive carcinogenesis, including oestrogen overproduction and hyperinsulinemia.^5^ Increased adiposity levels stimulate a pro-inflammatory state with dysregulated immune responses leading to increased production of adipokines and cytokines, including altered levels of IGF-1, IL-6, TNF alpha, C reactive protein and leptin.^6^ Despite the evidence that obesity increases the risk of developing cancer for many tumour types, some studies have suggested that high BMI may be associated with improved survival for some cancers treated with systemic anti-cancer therapy (SACT), a phenomenon referred to as the obesity paradox.^7-11^ However, there has been some contradictions reported in the literature, for example, obesity has been previously shown to be associated with worse outcomes in patients with resected early stage malignant melanoma, however the contrary has been demonstrated in obese patients with unresectable advanced stage malignant melanoma, receiving immune checkpoint inhibitors and oral targeted therapies.^11,12^

Renal cell carcinoma (RCC) accounts for 4% of cancer diagnoses in the United Kingdom, with clear cell histology making up the majority of cases and the prevalence is increasing.^13^ Obesity is a proven modifiable risk factor for the development of RCC.^14^ Outcomes remain poor, with 5-year survival in patients with metastatic disease of 14%.^15^ Patients with newly diagnosed metastatic clear cell RCC are predominantly treated with oral tyrosine kinase inhibitors (TKIs) that target vascular endothelial growth factor (VEGF) and immune checkpoint inhibitors.^16^ Previous observational studies in patients with RCC treated with either TKIs or immune checkpoint inhibitors, have demonstrated a high BMI to be associated with improved outcomes.^14,17,18^ This study reviewed the impact of BMI in patients with clear cell RCC receiving a range of treatments including TKIs and immune checkpoint inhibitors, using data from the National Health Service (NHS), England.

## Methods

We investigated the association between BMI and survival in patients with advanced RCC receiving systemic treatment using patient data provided by NHS England. The Systemic Anti-Cancer Therapy database is a population-based resource of SACT activity reported routinely by NHS trusts in England.^19^ These data included treatment intent, performance status, age, sex, height, weight and the Charlson co-morbidity score within all NHS institutions across England.^19^ We performed a retrospective analysis of the SACT database conforming to STROBE guidelines.^20^

BMI is defined as the body mass divided by the square of the body height, expressed in units of kg/m^2^. The association between BMI (BMI: ≥25 kg/m^2^ and BMI: <25 kg/m^2^) on survival following treatment with targeted therapy and immunotherapy was investigated. Survival from commencement of first line therapy with TKI therapy (sunitinib, pazopanib, axitinib, cabozantinib and tivozanib), MTOR inhibitors (everolimus and temsirolimus) and immune checkpoint inhibitors (ipilimumab and nivolumab) was estimated.

### Statistical Analysis

In the survival analysis, the survival curves for patients with BMI <25 kg/m^2^ and ≥25 kg/m^2^ were estimated using the life table method, which divided the follow-up time since the first administration into 3-month intervals and calculates survival probabilities at each interval. The ratios of annual death rates were estimated using Poisson regression and adjusted for year of diagnosis, age at diagnosis, sex, grade, performance status, Charlson score and prior nephrectomy. Patients were excluded from the analysis if data on height or weight was incomplete or if enrolled on a therapeutic clinical trial. Indicators for missingness were created for tumour grade, performance and Charlson score, allowing the model to account for missing data.

## Results

**Figure 1.**
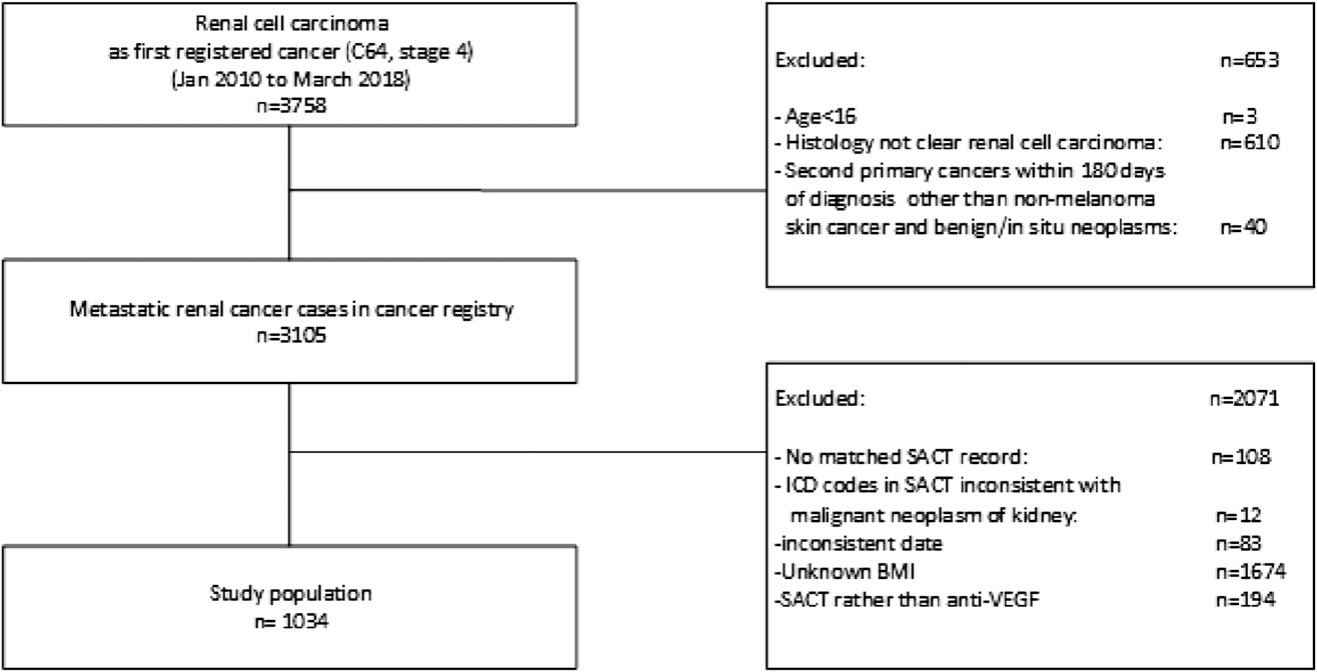
Derivation of study population.

From January 2010 to March 2018, 3758 patients were registered on the database with RCC (Figure 1). Of these, 2724 patients were excluded from the analysis. The most common reason for exclusion being non-clear cell histology subtypes (n = 610) and incomplete BMI data (n = 1674). In total, data from 1034 patients were used in the analysis.

Patients included were predominantly male (72% vs 28%), with a performance status of 0 to 1 (87%) and a Charlson co-morbidity score of 0 (84%) (Table 1). A Charlson score of 0, indicating the absence co-morbidities was more common in patients with a BMI <25 kg/m^2^ compared to patients with a higher BMI ≥25 kg/m^2^ (87.9% vs 81.2% respectively, *P* .02). The majority of patients (n = 1003) received treatment with oral TKI therapy targeting VEGF, with 53.3% of patients receiving treatment with pazopanib and 43.7% receiving treatment with sunitinib. 3% of patients (n = 31) received treatment with other agents including immune checkpoint inhibitors.

**Table 1.** Characteristics of 1034 Patients With Metastatic Renal Cancer.

	Body Mass Index		*P*	Total
	<25 kg/m^2^	≥25 kg/m^2^		
Total	355	679	—	1034
Year of diagnosis				
2010-11	9 (2.5)	21 (3.1)	.55	30 (2.9)
2012-13	50 (14.1)	117 (17.2)		167 (16.2)
2014-15	115 (32.4)	213 (31.4)		328 (31.7)
2016-18	181 (51.0)	328 (48.3)		509 (49.2)
Age at diagnosis				
<50	37 (10.4)	54 (8.0)	.07	91 (8.8)
50-59	100 (28.2)	197 (29.0)		297 (28.7)
60-69	112 (31.5)	266 (39.2)		378 (36.6)
70-79	91 (25.6)	139 (20.5)		230 (22.2)
80+	15 (4.2)	23 (3.4)		38 (3.7)
Sex				
Male	236 (66.5)	499 (73.5)	.02	735 (71.1)
Female	119 (33.5)	180 (26.5)		299 (28.9)
Grade				
I	6 (3.2)	13 (3.3)	.43	19 (3.3)
II	39 (20.7)	104 (26.5)		143 (24.7)
III	86 (45.7)	174 (44.4)		260 (44.8)
IV	57 (30.3)	101 (25.8)		158 (27.2)
Unknown	167	287		454
Performance				
0: no restriction	62 (28.3)	117 (31.6)	.58	179 (30.4)
1	124 (56.6)	206 (55.7)		330 (56.0)
2+	33 (15.1)	47 (12.7)		80 (13.6)
Unknown	136	309		445
Charlson score				
0: No comorbidities	304 (87.9)	543 (81.2)	.02	847 (83.4)
1	29 (8.4)	81 (12.1)		110 (10.8)
2+	13 (3.8)	45 (6.7)		58 (5.7)
Unknown	9	10		19
Surgery				
Nephrectomy	244 (68.7)	412 (60.7)	.01	656 (63.4)
No	111 (31.3)	267 (39.3)		378 (36.6)
Targeted anti-VEGF				
Pazopanib	176 (49.6)	375 (55.2)	.14	551 (53.3)
Sunitinib	165 (46.5)	287 (42.3)		452 (43.7)
Other	14 (3.9)	17 (2.5)		31 (3.0)
Vital status				
Alive	98 (27.6)	234 (34.5)	.03	332 (32.1)
Dead	257 (72.4)	445 (65.5)		702 (67.9)

**Figure 2.**
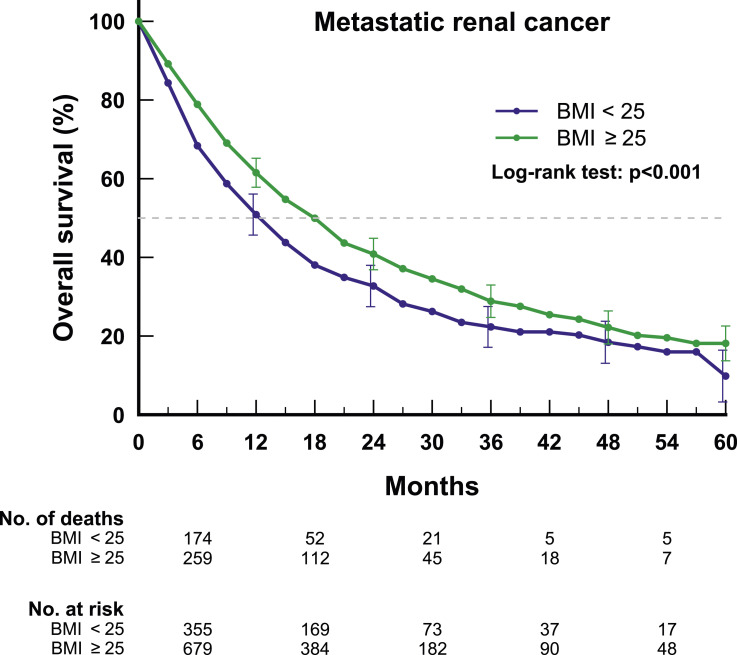
Overall survival of patients with BMI ≥25 kg/m^2^ vs patients with BMI <25 kg/m^2^. *Median survival time <25 kg/m^2^: 12.6 months (10.1-14.4); >=25 kg/m^2^: 17.9 months (15.4-20.0).

**Figure 3.**
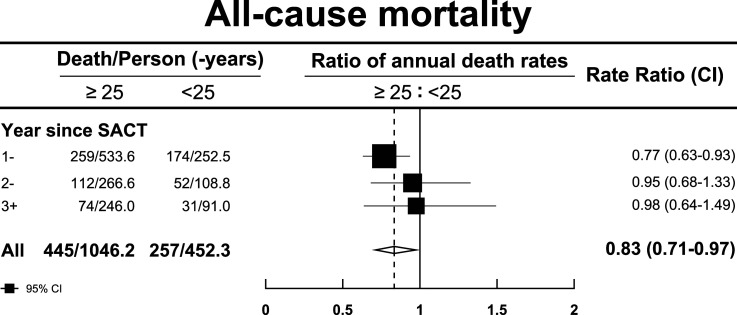
Ratio of annual death rates in patients with BMI ≥25 kg/m^2^ vs patients with BMI <25 kg/m^2^ by year since SACT (poisson regression model adjusted by age, sex, year of diagnosis, grade and performance).

Median overall survival for patients with a BMI <25 kg/m^2^ was 12.6 months (95% CI; 10.1-14.4) vs 17.9 months (15.4-20.0) for patients with BMI ≥25 kg/m^2^ (*P* < .001) (Figure 2). The association between BMI and improved survival was greatest in the first year of commencing SACT, with an adjusted mortality rate of 68.9% for patients with BMI <25 kg/m^2^ compared to 48.6% for patients with BMI ≥25 kg/m^2^ (rate ratio .77, .63 to .93) (Figure 3). The association between BMI and outcomes in patients receiving SACT for longer than 1 year was not significant. Treatment with different types of oral TKIs did not impact the effect of BMI on outcome (*P* .14). All-cause mortality adjusted for age, sex, year of diagnosis, grade and performance status was lower in patients with BMI ≥25 kg/m^2^ compared to patients with BMI <25 kg/m^2^, 42.6% vs 56.8% (ratio .83, 95% CI .71 to .97).

## Discussion

Obesity rates are increasing globally, as are rates of new cancer diagnoses.^3^ Obesity and its link with carcinogenesis is well documented.^3^ Elevated blood leptin levels, oestrogen and hyperglycaemia all play a role in the development of cancer.^6^ Circulating cytokines IL-6 and insulin like growth factors also play a part in allowing cancer cells to divide and proliferate uncontrollably.^21^ Obesity is a known modifiable risk factor for the development of kidney cancer but paradoxically, some obese patients demonstrate a better response to SACT.^22^ This has been demonstrated in melanoma and kidney cancer trials, where patients treated with SACT had better outcomes if they were defined as obese.^11,23^ TKIs have been shown to mediate glucose metabolism by causing tumour-induced hypoglycaemia from overexpression of insulin-like growth factor II on tumour cells.^18,24^ Obesity leads to a pro-inflammatory environment with chronic cytokine activation, potentially promoting the effect of certain drug treatments in relation to the tumour microenvironment.^25^ Obesity is also associated with an increased level of T cells within fatty adipose tissue suggesting an enhanced immunogenic environment in obese patients.^26-28^

In this study, we demonstrated that high BMI was significantly associated with improved survival in patients with metastatic clear cell RCC receiving treatment with SACT. The impact of BMI on outcome was most significant during the first year of treatment. A low BMI can be indicative of higher grade, higher stage and poor prognostic disease at diagnosis.^22^ This is reflected by the increased mortality seen in this study in the first year in low BMI patients, compared to patients with BMI ≥25 kg/m^2^.

Obesity is an independent risk factor for the development of cancer and other co-morbidities including diabetes and cardiovascular disease. A Charlson score of zero, indicating no co-morbidities, was more frequent in patients with a normal or low BMI, confirming that obese patients have more associated comorbidities. Cytoreductive surgery was also more likely to be performed in patients a normal or low BMI, suggesting obese patients are less likely to be surgically operable due to co-morbidities and worse post-operative outcomes.

Limitations of this study included incomplete data, as the SACT database requires BMI data to be inputted in order to prescribe therapies based on weight and body surface area, however this is increasingly not required for SACT used in RCC, where a standardised dose is prescribed regardless of BMI. Similarly, histological subtyping and prognostic risk scoring such as the International Metastatic Renal Cell Carcinoma Database Consortium (IMDC) criteria would have aided further sub-analysis, however this data are not routinely collected. It was not possible to define any link with ethnicity due to the number of patients involved. Lastly, retrospective studies come with increased risk of confounding errors that may exert influence on outcomes.^29^

Baseline BMI at diagnosis as a surrogate marker of obesity does not reflect the dynamic weight changes often seen in cancer patients in the acute phase of their illness.^30^ A meta-analysis by Graff et al using cumulative average BMIs rather than BMI at diagnosis determined that significant weight changes at diagnosis, measured using BMI prior to cancer diagnosis compared to a BMI post-diagnosis, was associated with a significantly increased risk of death.^23^ Cancer cachexia can be more accurately measured by the loss of muscle mass (sarcopenia), which itself is a poor prognostic sign.^31^ Capturing sarcopenia data has been suggested as a future biomarker in developing clinical trials.^32^

T cells plays a key role in the interaction of immunity, metabolism and response to treatments particularly in the era of immune checkpoint inhibitors, and there is further evidence that genes associated with insulin resistance markers are upregulated in obesity.^33,34^ Increased leptin expression has been demonstrated to play a role in PD-1 expression in tumour cells, supporting the hypothesis that obesity develops a pro-inflammatory state which may improve responses to treatment.^35^ Immune checkpoint inhibitors such as nivolumab and pembrolizumab are now predominantly used in the current treatment of both melanoma and kidney cancer by targeting the PD-1/PDL-1 pathway.^36,37^ The current management of advanced kidney cancer has advanced from single agent therapy towards a combination of immune checkpoint inhibitor and TKI therapy and the impact of BMI on these treatment strategies is currently unclear.^38^ Furthermore, the association of obesity on newly approved agents such as belzultifan, a hypoxia-inducible factor (HIF) 2α inhibitor and the use of the checkpoint inhibitors in the adjuvant setting post nephrectomy for kidney cancer is also unknown.^39,40^

## Conclusion

This study demonstrated an improved survival for patients with advanced clear cell RCC and BMI ≥25 kg/m^2^, compared to those with BMI <25 kg/m^2^, receiving various types of systemic treatment. Obesity continues to be an ongoing problem for health services globally and will have an ongoing impact on the delivery of cancer care for the foreseeable future. Improved survival in obese patients may be associated with a better response to systemic therapies. Further prospective studies investigating the impact of body composition on survival outcomes are needed.

## Supplemental Material


Supplemental Material - The Impact of Body Mass Index (BMI) on Clinical Outcomes for Patients Receiving Systemic Anti-Cancer Therapies for Advanced Clear Cell Renal Carcinoma
Supplemental Material for The Impact of Body Mass Index (BMI) on Clinical Outcomes for Patients Receiving Systemic Anti-Cancer Therapies for Advanced Clear Cell Renal Carcinoma by John Greene, Zhe Wang, Benjamin Harris, David Dodwell, and Simon Lord in Journal of Cancer Control.
